# RF‐Shielding of Laser‐Cut Venous Stents: Calculations, Simulations, and Experiments

**DOI:** 10.1002/mrm.70207

**Published:** 2025-11-30

**Authors:** Lisa Regler, Felix Spreter, Kian Tadjalli Mehr, Niklas Verloh, Klaus Düring, Wibke Uller, Michael Bock, Simon Reiss

**Affiliations:** ^1^ Division of Medical Physics, Department of Diagnostic and Interventional Radiology, University Medical Center Freiburg, Faculty of Medicine University of Freiburg Freiburg Germany; ^2^ Department of Diagnostic and Interventional Radiology, University Medical Center Freiburg, Faculty of Medicine University of Freiburg Freiburg Germany; ^3^ Alaxo GmbH Wallgau Germany

**Keywords:** *B*_1_ calculations, MRI artifacts, RF shielding, venous stents

## Abstract

**Purpose:**

To develop an analytical model of the RF‐shielding of laser‐cut venous stents for different orientations and stent geometries.

**Methods:**

Laser‐cut venous stents are modeled as a grid composed of circular and rectangular loops. As these loops are orthogonal they shield different components of the transmit RF field. The shielding is calculated using Faraday's induction law, taking into account the self‐inductance and mutual coupling of circular and rectangular loops, and Biot–Savart's law. The shielding of 95 stent‐mimicking models was calculated, and numerical simulations and measurements were performed at both 1.5 and 3T for validation.

**Results:**

The shielding of circular and rectangular loops differed by less than 3.3% and 10% between calculations, simulations, and measurements. The overall stent length had negligible impact on the shielding (< 8%). The combined shielding of both loop types showed deviations of less than 10% compared to stent models with varying cell sizes. Orienting the stents at an oblique angle other than 0° or 90° to *B*_0_, the model‐predicted shielding values deviated by less than 14%/10% from the measured values at 1.5 T/3 T.

**Conclusion:**

The model allows for the calculation of the *B*_1_ shielding of laser‐cut venous stents depending on their geometry and orientation relative to *B*_0_. Thus, it may help to adapt imaging parameters such as the flip angle to optimize monitoring of stent patency.

## 1 Introduction

Classical endovascular stenting procedures target the arterial system, where the placement of a stent is often necessary to reopen an obstructed arterial blood vessel to reestablish blood flow. Endovascular stenting has also emerged as a favorable treatment option for venous compression syndromes [1]. Initially, these venous treatments were performed with the same stents as in the artery resulting in suboptimal outcomes since the low‐pressure system of the veins requires stents with different mechanical properties than the high‐pressure arteries. Recently, dedicated venous stents have become commercially available that are optimized for venous applications—these devices exhibited encouraging primary and secondary patencies [2, 3]. Laser‐cut venous stents have been designed with different geometries and mechanical properties; an important parameter in these designs is the radial strength that must be sufficiently high to assure optimal wall apposition but should be low enough to preserve the flexibility of the vein [4, 5].

In venous stents cumulative in‐stent restenosis rates of 5% have been reported at 72 months [6] after implantation which necessitates noninvasive imaging to monitor stent patency. Magnetic resonance venography (MRV) is established as a favorable imaging method to both diagnose and follow‐up compression syndromes [7, 8]. However, the metallic structure of the stent can reduce the MRV signal in the stent lumen by susceptibility artifacts and radiofrequency (RF) shielding. Venous stents are commonly made from Nitinol (NiTi), a material that has a similar magnetic susceptibility to the surrounding tissue so that the signal voids do not extend more than 2–3 mm above the surface of stents [9, 10, 11, 12]. In contrast, RF shielding can substantially reduce the image quality in stents as the *B*_1_ field is reduced due to RF eddy currents [12, 13] which can reduce the intraluminal signal by up to 70% limiting the ability of MRV to image a potential in‐stent stenosis [14]. As the RF shielding affects both the local flip angle during RF excitation and the received signal during data acquisition, adapting imaging parameters may help to partly restore the intraluminal signal [15].

It was also shown that inner‐stent signal is independent of *B*_0_ [16, 17]. Previous studies demonstrated that the induced RF field *B*_1ind_ strongly depends on the number of struts forming the stent—that is, the cell size and the orientation to *B*_0_ [10, 12, 18, 19, 20, 21, 22, 23]. Bouillot et al. [12], however, reported a low correlation solely to the number of wires. Ohno et al. [21] investigated the signal decrease as a function of the cell size of various stent designs, but without discussing the effect of the orientation of the *B*_1_ field. Orientation effects were assessed in more detail in studies [14, 24, 25] that found that the shielding in stents parallel to *B*_0_ depends linearly on the stent cell width. When stents were oriented orthogonal to *B*_0_, however, a clear relationship between shielding and the cell width or length could not be found. A comprehensive study of the shielding of stents for all possible orientations and geometries is missing so far; however, it is important to adapt the flip angle depending on the individual stent shielding. This study focuses on laser‐cut venous stents as they showed homogeneous inner‐stent shielding compared to braided venous stents [25].

In this work analytical and numerical models are developed to calculate the shielding depending on their cell geometry and orientation relative to the *B*_1_ field. These results are compared to simulations and measurements of stent models and a set of commercially available venous stents at both 1.5 and 3 T.

## 2 Methods

### 2.1 Stent Model

In whole‐body MRI systems, typically quadrature transmit coils (body coils) are used to create a circularly polarized *B*_1_ field for RF excitation that is orthogonal to *B*_0_ [26]. A circular polarization is preferred over linear polarization as it decreases inhomogeneity artifacts while increasing efficiency [27]. In general, this *B*_1_‐field can be described as [28]:

(1) $${\vec{B}}_{1}\left(t\right)=\left(\begin{array}{l} B_{1x}\left(t\right)\\ B_{1y}\left(t\right)\cdot e^{-i\frac{\pi }{2}}\\ 0 \end{array}\right)e^{-i\omega t}$$

As the bandwidth of the RF excitation pulses is typically five orders of magnitude smaller than the Larmor frequency ω, a harmonic time‐dependency is assumed and the small frequency modulation during the pulse is ignored.

The presence of a stent leads to a shielding of *B*_1_ due to the interaction of *B*_1_ with the metallic structure of the stent—this interaction can be calculated separately for *B*_1*x*_ and *B*_1*y*_ due to the superposition principle. According to the Faraday's law of induction, the RF field induces a voltage *U* in the stent wires due to the changing magnetic flux Ф (see next section):

(2) $$U=-\frac{d}{dt}Ф=-\frac{d}{dt}\oint_{\mathrm{dA}}\vec{E}d\vec{l}=-\frac{d}{dt}\int_{A}{\vec{B}}_{1}d\vec{A}=-\int_{A}\left(\frac{\partial }{\partial t}{\vec{B}}_{1}\right)d\vec{A}$$

This voltage *U* drives a current that is only induced in loops whose surface normal vectors $d\vec{A}$ have nonvanishing projections onto ${\vec{B}}_{1}$. The currents create magnetic fields ${\vec{B}}_{1\mathrm{ind}}$ that change the incident RF field to: ${\vec{B}}_{1\mathrm{tot}}\left(x,y,z\right)={\vec{B}}_{1}+{\vec{B}}_{1\mathrm{ind}}\left(x,y,z\right)$. The minus sign in Equation (2) leads, according to Lenz's law, to an opposed sign of ${\vec{B}}_{1\mathrm{ind}}$ inside the stent, and thus, to a shielding of the *B*_1_ field. Hewett and Hewitt [29] showed that if the wavelength of the incident field is larger than the distance of wires, the field follows the near‐field behavior and the Laplacian boundary conditions apply. Thus, the *B*_1_ field is assumed to be quasi‐static and the interaction between the stent and the *B*_1_ field is dominated by inductance.

**FIGURE 1:**
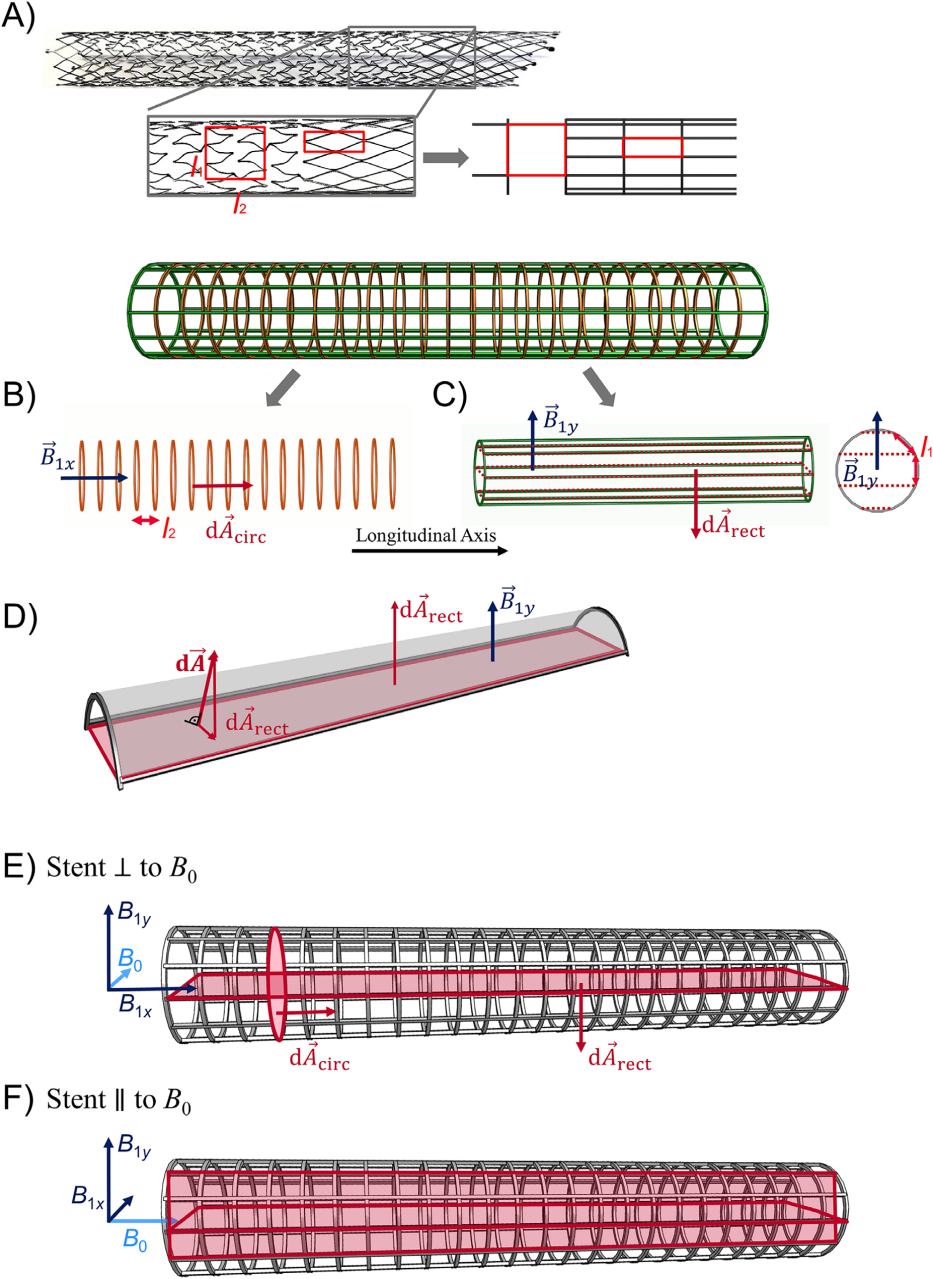
(A) A commercial laser‐cut venous stent. It consists of a complex grid with two segments of different cell geometry. In this work, the cells are simplified by rectangulary cells width *l*_1_ and length *l*_2_. (B) The stents are decomposed into circular loops (orange, distance *l*_2_) and (C) rectangular loops (green). The latter are formed by *N* bars that are parallel to the stent's long axis and distributed circumferentially with distance *l*_1_, as well as two circular loops at both ends of the stent. (D) Due to the dependence of the induced current on the scalar product of surface normal vector $d\vec{A}$ and the incident *B*_1_, the bent rectangle is projected onto a plane orthogonal to *B*_1_. (E) The surface normal vectors are defined by $d{\vec{A}}_{\text{circ}}$ and $d{\vec{A}}_{\text{rect}}$ for circular and rectangular loops, respectively. In case the stent perpendicular to *B*_0_, the *B*_1*x*_ and *B*_1*y*_ components of a circularly polarized *B*_1_ field are perpendicular to the circular and rectangular loops, respectively. (F) If the stent is parallel to *B*_0_, both *B*_1*x*_ and *B*_1*y*_ are shielded by rectangular loops.

Figure 1A shows a commercial laser‐cut venous stent with a complex grid pattern. The grid is typically described by its cell design; in this work, the cell width orthogonal to the stent's long axis is denoted *l*_1_ and the cell length parallel to the long axis *l*_2_. This repetitive structure can be modeled as closed circles and rectangles along which loop currents can be induced as depicted in Figure 1B,C. These circular (orange) and rectangular (green) loops with surface normal vectors $d{\vec{A}}_{\text{circ}}$ and $d{\vec{A}}_{\text{rect}}$ are orthogonal to each other, and combining them yields the complete grid structure. Stents composed by both loops will be referred to as “grid stents” in this work. Both loops shield two independent *B*_1_ components, since any arbitrarily oriented magnetic field can be decomposed into one component parallel to $d{\vec{A}}_{\text{circ}}$ and in another parallel to $d{\vec{A}}_{\text{rect}}$. To calculate Ф of rectangles, $d{\vec{A}}_{\text{rect}}$ (red surface) is used which results from the projection of $d\vec{A}$ of rectangles along ${\vec{B}}_{1y}$ (Figure 1D). To calculate the current within each cell, it is sufficient to consider only the current along the edges of rectangular loops as currents cancel in common wire segments of $d\vec{l}$ of cells parallel to the long axis.

The stent orientation relative to *B*_0_ determines which *B*_1_ component is shielded by the circular and rectangular loops. Here, two cases are described: orthogonal and parallel orientation of the stent long axis to *B*_0_. Other orientations can be formed by the superposition of these cases. In the first case *B*_1*x*_ is perpendicular to the circular loops, and *B*_1*y*_ is perpendicular to the rectangular loops. Consequently, *B*_1*x*_ is only shielded by the circular loops whereas *B*_1*y*_ is only shielded by the rectangular loops. In the second case, both the *B*_1*x*_ and *B*_1*y*_ components are perpendicular to the rectangular loops due to the rotational symmetry of the stent. Consequently, both components are shielded similarly by the rectangular loops.

Commercial stents commonly (except, e.g., sinus‐Obliquus, optimed, Germany) have one‐cell design with a given cell size. Thus in this study, the circular loops with radius *R* have a constant distance which is the cell length *l*_2_. A typical venous stent radius of *R* = 8 mm was chosen. The number of circular loops *m* was chosen such that the stent length is *L* = (*m* − 1)*l*_2_ = 100 mm. The rectangular loops vary in distance and width and are constructed from *N* longitudinal bars with length *L* that are distributed along the circumference with distance *l*_1_.

### 2.2 Calculation of *B*_1_ ‐Shielding

If the stents act as a perfect electric conductor (PEC), the current *I* in the loop is given by [30]:

(3) $$U=-L\frac{dI}{dt}$$

Combining (2) and (3) yields

(4) $$-L\int \frac{dI}{dt}\mathrm{dt}=-\int \frac{dФ}{dt}\mathrm{dt}\to Ф\left(t\right)=L\;I\left(t\right)$$

To calculate the induced field in the stent mutual coupling between individual loops needs to be taken into account. For two coupled loops, the mutual inductance *M*_21_ can be calculated using Neumann's formulation [28]:

(5) $$M_{21}=\frac{\mu_{0}}{4\pi }\oint \oint \frac{d{\vec{l}}_{1}d{\vec{l}}_{2}}{{\vec{r}}_{1}-{\vec{r}}_{2}}$$

**FIGURE 2:**
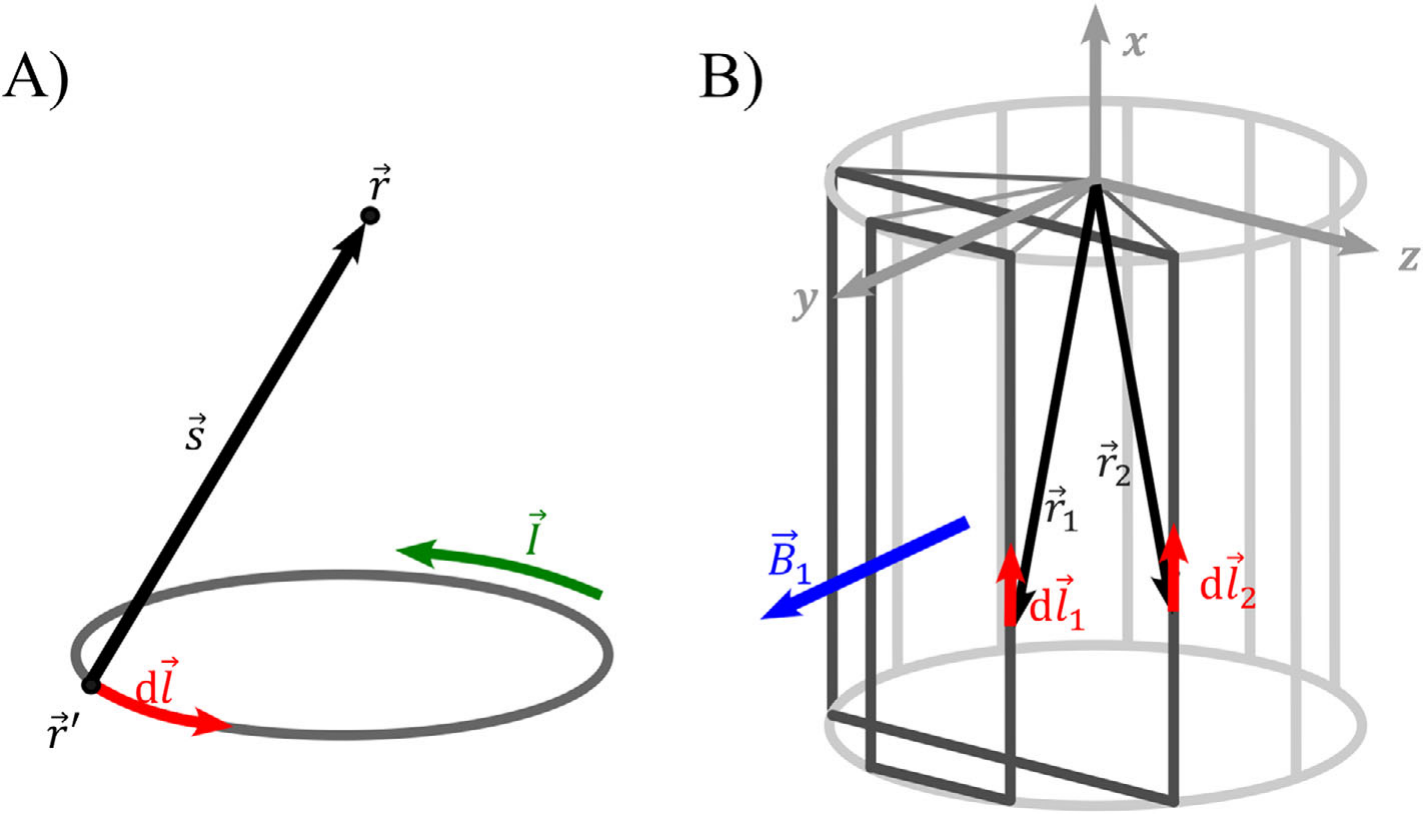
Visualization of the parameters for the calculation of the RF shielding. (A) A current $\vec{I}$ at position $\vec{r}$' induces a magnetic field at point $\vec{r}$. (B) Visualization of the calculation of mutual inductance of rectangular loops. It is assumed that ${\vec{B}}_{1}$ is parallel to the surface normal vectors of the rectangular loops.

$d{\vec{l}}_{1}$ and $d{\vec{l}}_{2}$ are the infinitesimal current elements at position ${\vec{r}}_{1}$ and ${\vec{r}}_{2}$ (Figure 2B). The voltages $U_{1}$ and $U_{2}$ in the two loops can be written as [28]:

(6) $$U_{1}=-L_{1}\frac{{dI}_{1}}{dt}-M_{12}\frac{{dI}_{2}}{dt}\;\text{and}\;U_{2}=-L_{2}\frac{{dI}_{2}}{dt}-M_{21}\frac{{dI}_{1}}{dt}$$

As *M*_21_ is equal to *M*_12_ [28], this results in a system of linear equations for multiple loops [31]:

(7) $$-U_{i}=L_{i}\frac{{dI}_{i}}{dt}+\sum_{i\neq j}M_{\mathrm{ij}}\frac{{dI}_{j}}{dt}$$

Here one can write $\underline{L}=L+M$ as a matrix, L being diagonal, and $\Phi =\sum {Ф}_{i}\;{\vec{e}}_{i}$ as vector with the magnetic flux for all loops, *i*. Finally, the induced current *I*_*i*_ in each loop i induces a magnetic field *B*_1ind_ at the position $\vec{r}$ according to Biot–Savart:

(8) $${\vec{B_{1}}}_{\mathrm{ind}}\left(\vec{r}\right)=\frac{\mu_{0}}{4\pi }I\oint \frac{d\vec{l}\;\times \;\hat{s}}{s^{2}}$$

Here $\vec{s}=\vec{r}-{\vec{r}}^{\prime }$, ${\vec{r}}^{\prime }$ is the position of the current element, and $d\vec{l}$ the normalized orientation of the current element (Figure 2A).

#### 2.2.1 Circular Loops

For a circular loop the flux ${Ф}_{c}$ is given by

(9) $${Ф}_{c}=\int_{A}{\vec{B}}_{1}rd\varphi dr\hat{x}=\pi R^{2}B_{1}$$

and the self‐inductance $L_{c}$ is [32]

(10) $$L_{c}=\mu_{0}R\left(\ln\left(\frac{8\;R}{\rho }\right)-2\right)$$

with the wire radius *ρ* and the magnetic permeability $\mu_{0}$. The mutual inductance *M*_21_ of two circular loops is [33]:

(11) $$M_{21}=\mu_{0}R\left(\left(\frac{2}{k}-k\right)F\left(k\right)-\frac{2}{k}E\left(k\right)\right);k=\frac{2R}{\sqrt{{\left(2R\right)}^{2}+d^{2}}}$$

with modulus *k*, *F*(*k*), and *E*(*k*) the complete elliptic integrals to modulus *k*, that describe the arclength of an ellipse and the compressed surface covered along the arc, and *d* the loop distance. The formula was calculated for all combinations of circular loops *i* and *j* ⋲ [1;*m*], *i* ≠ *j*. Thus, $\underline{L}=L_{c}\;1+M$ has size *m* × *m*. The current in each loop *I*_i_ is solved from the linear Equation (7) using $I=-\underline{L}\setminus \Phi$ with the built‐in operator of MATLAB. A 2D map of ${\vec{B_{1}}}_{\mathrm{ind},i}$ (central slice) was numerically calculated for each loop *i* with Equation (8) by discretizing ${\vec{r}}^{\prime }$ in 100 segments. The induced field of all loops was obtained by ${\vec{B_{1}}}_{\mathrm{ind}}\left(\vec{r}\right)=\sum_{i}{\vec{B_{1}}}_{\mathrm{ind},i}\left(\vec{r}\right)$.

#### 2.2.2 Rectangular Loops

For a rectangular loop the self‐inductance is [34]:

(12) $$\begin{array}{ll} L_{i} & =\frac{\mu_{0}}{4\pi }4\left[\left(L+w_{i}\right)\log\left(\frac{2L\;w_{i}}{\rho }\right)-L\log\left(L+b_{i}\right)\right.\\ & \;\left.-w_{i}\log\left(w_{i}+b_{i}\right)-\frac{7}{8}\left(L+w_{i}\right)+2\left(b_{i}+\rho \right)\right] \end{array}$$

*L* and *w*_i_ are the length and width of loop *i*, with $w_{i}=2R\sin\left(\gamma_{i}\right)$, *γ*_i_ = (*i* − $\frac{1}{2}$)(2π/*N*), $b_{i}=\sqrt{L^{2}+{w_{i}}^{2}}$ and ρ the wire radius. The mutual inductance of rectangles was calculated numerically using Equation (5). Both position vectors ${\vec{r}}_{i}$ and ${\vec{r}}_{j}$ were discretized in 100 segments along each of the four wire segments. The ceil (*N*/2) bar was omitted for models with odd numbers of bars, that is, only separate loops are considered as the *1*/(*r*_1_–*r*_2_) term in *M* would diverge when combining the additional bar with a neighboring bar (ceil (*N*/2) + 1 or ceil (*N*/2) − 1) that already formed a rectangular loop.

As ${\vec{B}}_{1}$ is parallel to $d{\vec{A}}_{i}$ the flux ${Ф}_{i}$ was calculated as ${Ф}_{i}$ = *B*_1_ *A*_rect,i_ = *B*_1_ *w*_i_ *L*. The current *I*_i_ for each rectangle was calculated solving $I=-\underline{L}\setminus \Phi$ as for the circular loops. ${\vec{r}}^{\prime }$ was discretized in 100 segments and ${\vec{B_{1}}}_{\mathrm{ind}}\left(\vec{r}\right)=\sum_{i}{\vec{B_{1}}}_{\mathrm{ind},i}\left(\vec{r}\right)$.

For more information, the reader is referred to the provided code [35].

Maps of *B*_1ind_ were calculated for stent models with circular loops with varying *l*_2_ from 0.6 to 30 mm, and for rectangular loops with *N* between 3 and 30, all with ρ = 0.2 mm. This was repeated for three typical stent lengths of *L* = 60, 80, and 100 mm. For the rectangular loops, *B*_1ind_ in the center (*x,y,z* = 0) was extracted. The calculated shielding maps of circular loops, however, showed an inhomogeneous shielding in the loops. Thus, *B*_1ind_ was averaged along the longitudinal axis of the central loop to the midpoint (*l*_2_/2) of its nearest neighbor. For each model the shielding *β* was quantified as the relative induced *B*_1_ field as *β* = *B*_1ind_/*B*_1_.

To assess the shielding and the shielding maps of grid stents, *β* and *B*_1ind_ were calculated as the sum of the shielding and ‐maps of the circular (*β*_circ_, *B*_1ind,circ_) and rectangular (*β*_rect_, *B*_1ind,circ_) loops:

(13) $$B_{1\mathrm{ind}}=\frac{1}{2}\left(B_{1\mathrm{ind},\text{rect}}\left(N\right)+B_{1\mathrm{ind},\text{circ}}\left(l_{2}\right)\right);\beta =\frac{1}{2}\left(\beta_{\text{rect}}\left(N\right)+\beta_{\text{circ}}\left(l_{2}\right)\right)$$

This was done for combinations of *N* = 4, 5, 6, and 10, and *l*_2_ = 5, 6.67, 10, and 20 mm.

To demonstrate that the model can be used to calculate the shielding of stents at oblique angles (20°, approx. 45° and 70°), stents were oriented in the coronal plane at an arbitrary angle δ relative to *B*_0_. Equation (13) was expanded to:

(14) $$\begin{array}{ll} B_{1\mathrm{ind}} & =\frac{1}{2}\left(B_{1\mathrm{ind},\text{rect}}\left(N\right)+\cos^{2}\left(\delta \right)B_{1\mathrm{ind},\text{rect}}\left(N\right)+\sin^{2}\left(\delta \right)B_{1\mathrm{ind},\text{circ}}\left(l_{2}\right)\right);\\ \beta & =\frac{1}{2}\left(\beta_{\text{rect}}\left(N\right)+\cos^{2}\left(\delta \right)\beta_{\text{rect}}\left(N\right)+\sin^{2}\left(\delta \right)\beta_{\text{circ}}\left(l_{2}\right)\right) \end{array}$$

Here the *y*‐component *B*_1*y*_ is only shielded by rectangular loops, whereas the *x*‐component *B*_1*x*_ is shielded by both, rectangular and circular loops.

### 2.3 Simulations

Finite‐difference‐time‐domain (FDTD) simulations of the RF‐induced *B*_1_ field were performed with the software package Sim4Life (v8.2.0, ZürichMedTech AG) for 32 models with only circular and 15 models with only rectangular loops as well as 28 grid stents. The models (*R* = 8 mm and *L* = 100 mm; including 80 and 60 mm for circular and rectangular loops) were constructed with *ρ* = 0.2 mm. The models were simulated as PECs that were immersed in a water bath (9 cm × 9 cm × 18 cm). Close to the loops, a spatial resolution of up to 0.2 × 0.2 × 0.2 mm^3^ was chosen, and elsewhere 1 × 1 × 1 mm^3^, resulting in approximately 4–10 × 10^6^ voxels per model.

Plane waves with frequencies of *f*_Larmor_ = 64 MHz and 123 MHz corresponding to *B*_0_ = 1.5 and 2.89 T were used as sources. The orientation of *B*_1_ was set orthogonal to the loops, that is, in the *x* direction for the circular loops and in the *y* direction for the rectangular loops (Figure 1C). The amplitude of *B*_1_ was set to 1 H/m. The shielding was quantified via *β* = (*B*_1_ − *B*_1ROI_)/*B*_1_ where *B*_1ROI_ is the reduced magnetic field that was extracted from the *B*_1_ maps for the calculations. All models were simulated additionally with *L* = 8 and 6 cm. To investigate if the homogeneity of shielding maps varies with frequency, one model with only rectangular loops was simulated for frequencies ranging from 63.8 to 250 MHz. And, to investigate the effect of the omission of the additional bar on *β* in cases with odd numbers of bars, a rectangular loop with *N* = 5 was simulated with and without the ceil (*N*/2) bar.

To demonstrate that the rectangular loops only shield *B*_1*y*_ for circularly polarized *B*_1_ fields, simulations were done with a superposition of two orthogonal plane waves (*B*_1*x*_ = *B*_1*y*_ = 1 and *π*/2 phase‐shift). The orientation of *B*_1*x*_ and *B*_1*y*_ relative to the longitudinal axis of rectangular loops was chosen to simulate the case of the stent being orthogonal to *B*_0_ (Figure 1C). As $B_{1}=\sqrt{{B_{1x}}^{2}+{B_{1y}}^{2}}$ the shielding was assessed as:

(15) $$\beta =1-\frac{B_{1\mathrm{ROI},y}\;}{B_{1y}}=1-\frac{\sqrt{B_{1\mathrm{ROI}}^{2}-{\left(\frac{B_{1}}{\sqrt{2}}\right)}^{2}}}{\frac{B_{1}}{\sqrt{2}}}=1-\sqrt{2{\left(\frac{B_{1\mathrm{ROI}}}{B_{1}}\right)}^{2}-1}$$

Furthermore, a stent orientation parallel to *B*_0_ was simulated where the rectangular loops are orthogonal to both *B*_1_ components, and the circular loops do not contribute to the shielding β.

Stent models with a grid structure orthogonal to *B*_0_ were simulated by combining both loop types. Six grid stents were constructed with different [*N*, *l*_2_] configurations (Supporting Information S1) and *L* = 100 mm. A circularly polarized *B*_1_ field was applied such that *B*_1*x*_ is perpendicular to the circular loops and *B*_1*y*_ is perpendicular to the rectangular loops. The shielding of the grid stents with [*N*, *l*_2_] are compared to $\beta =\frac{1}{2}\left(\beta_{\text{rect}}\left(N\right)+\beta_{\text{circ}}\left(l_{2}\right)\right)$ obtained for rectangular loops with same *N* and circular loops with same *l*_2_.

### 2.4 Experiments

#### 2.4.1 Stent Models

The shielding of three different sets of stent models was measured: models with only circular loops, only rectangular loops, and grid stents. The circular loops with *R* = 8 mm and *ρ* = 0.2 mm were built from coated copper wires (Bürklin, Germany). The rings were enameled, which does not influence the magnetic field substantially. Furthermore, enameled copper wires were used due to limited availability reasons. It was not feasible to manufacture the loops with a commercial laser‐cut *ρ* = 0.1 mm. The loops were stacked over a length of 100 mm with distances *l*_2_ that varied from 2 to 30 mm in steps of 2 mm. The models with only rectangular loops (*ρ* = 0.2 mm, *L* = 100 mm) consisted of *N* noncoated copper wires (BKL Electronic, Germany) that were connected using two circular loops (*R* = 8 mm, *ρ* = 0.2 mm) at the ends. The straight wires were distributed circumferentially in equidistant steps (360°/*N*). Twelve models were constructed with *N* = 16, 14, 12, 11, 10, 9, 8, 7, 6, 5, 4, 3. Grid stents were soldered with six different configurations similar to the simulated models (Figure S1). Images of the models and phantom are provided in Figure S1.

#### 2.4.2 Measurements

Measurements were conducted at both, 1.5 T (Magnetom Aera, Siemens) and 3 T (PrismaFit, Siemens) using the body coil for transmission and a four‐channel Flex coil in combination with the spine array for signal reception. The stent models were placed at isocenter in a distilled water bath (700 mL) with 3 mL contrast agent Gadoteridol (ProHance) to shorten *T*_1_ to about 100 ms. All loops and grid stents were measured with their longitudinal axis in the coronal plane. The circular loops were measured with the longitudinal axis orthogonal to *B*_0_. The rectangular loops and grid stents were oriented both, orthogonal and parallel to *B*_0_. Two images were acquired of each model with GRE sequences (TR/TE: 200/3.87 ms, FOV: 180 × 125–180 × 180 mm^2^ (depending on stent orientation), Voxel size: 1.02 × 1.02 × 2.5 mm^3^, BW: 660 Hz [3 T] and 309 Hz [1.5 T]). The applied FA was *α*_1_ = 31.5° (1.5 T) and 40.6° (3 T) for the first image and the second image was acquired with *α*_2_ = 2*α*_1_. For rectangular loops parallel to *B*_0_ and the grid stents, the FA was increased by a factor of 2 to compensate for the strong shielding of these models [12] and to increase SNR.

#### 2.4.3 Evaluation

Apparent FA maps α(x,y) were calculated from the relative signal intensities *I*_1_ and *I*_2_ of the two images. The FLASH signal equation [36] yields:

(16) $$\frac{I_{1}}{I_{2}}=\frac{\sin\left(\alpha \right)}{\sin\left(2\alpha \right)}\frac{\left(1-\cos\left(2\alpha \right)E_{1}\;\right)}{\left(1-\cos\left(\alpha \right)E_{1}\;\right)}=\frac{1}{2\cos\left(\alpha \right)}\frac{\left(1-\cos\left(2\alpha \right)E_{1}\;\right)}{\left(1-\cos\left(\alpha \right)E_{1}\;\right)}$$

with $E_{1}=e^{-\frac{\mathrm{TR}}{T_{1}}}$. From the ratio $\frac{I_{1}\left(x,y\right)}{I_{2}\left(x,y\right)}$, flip angle maps α(x,y) were calculated using a look‐up table for Equation (16). To estimate *E*_1_, *T*_1_ of the water bath was measured via a series of GRE images with saturation recovery (*T*_I_ = 55, 70, 100, 125, 150, 175, 200, 250, 350, 500, 1000 ms) and a mono‐exponential fit using the fit equation $A\left(t\right)=A_{0}\left(1-e^{-\frac{\mathrm{TR}}{T_{1}}}\right)$.

The shielding maps *β*(*x,y*) were calculated from α(x,y) according to:

(17) $$\beta \left(x,y\right)=1-\frac{\alpha \left(x,y\right)}{\alpha_{\mathrm{BG}}\left(x,y\right)}$$

Here $\alpha_{\mathrm{BG}}\left(x,y\right)$ is the map of the background FA which was measured without any stent model. For rectangular and circular loops orthogonal to *B*_0_ only one *B*_1_ component is shielded. Assuming the amplitudes of the *B*_1*x*_ and *B*_1*y*_ component are equal, the background FA is $\alpha_{\mathrm{BG}}\left(x,y\right)=\alpha_{\mathrm{BG},x}\left(x,y\right)+\alpha_{\mathrm{BG},y}\left(x,y\right)=2\alpha_{\mathrm{BG},x}\left(x,y\right)$. Consequently, the shielding of one *B*_1_ component was calculated by:

(18) $$\beta \left(x,y\right)=1-\frac{\alpha \left(x,y\right)-\alpha_{\mathrm{BG},x,y}\left(x,y\right)}{\alpha_{\mathrm{BG},x,y}\left(x,y\right)}=1-\frac{2\alpha \left(x,y\right)-\alpha_{\mathrm{BG}}\left(x,y\right)}{\alpha_{\mathrm{BG}}\left(x,y\right)}$$

The shielding of each model was calculated from the mean value of β(x,y) in a small ROI at the center of the models.

The shielding of the grid stents orthogonal to *B*_0_ was compared to the combined shielding of the individually measured rectangular and circular loops as: $\frac{1}{2}$ (*β*_rect_(*N*) + *β*_circ_(*l*_2_)).

To validate the model for an arbitrary orientation, the shielding of grid stents was measured with an angle of δ = 20°, approx. 45° and 70° at both, 1.5 and 3 T, respectively.

The shielding of the stent models was also compared to 13 different commercially available NiTi venous stents which were measured at 1.5 and 3 T before [14, 24]. Further information about the brand, model, and geometry is given in the Figure S2. All stents have a radius of *R* = 8 mm and a strut radius of *ρ* = 0.1 mm, whereas *l*_*1*_ and *l*_*2*_ vary from 2 to 24 and 4 to 8.4 mm between different stent types. Further information about the brand, model, and geometry is given in the Figure S2.

To demonstrate that the known value of *β* can be used to restore the intraluminal flip angle, a measurement was performed with a commercial stent (*β* = 30%) in a venous phantom (United Biologics, USA) at 3 T. A GRE sequence (GRE: TR/TE = 7.4/4.7 ms, FOV: 300 × 271 mm^2^, Voxel size: 1 × 1 × 5 mm^3^, BW: 299 Hz) was acquired three times: first without the stent and a target flip angle of FA_target_ = 10° for reference, then with the stent (1 − *β* = 0.68) and an adapted flip angle of FA_adapted_ = $\frac{1}{1-\beta }$ FA_target_ = 15°. In this case, the intraluminal flip angle is expected to achieve the target value; however, the SNR is still reduced due to reciprocal shielding of the receive field by the ratio $\frac{{\mathrm{SNR}}_{\mathrm{ref}}\left(\mathrm{FA}=10^{{}^{\circ}}\right)}{{\mathrm{SNR}}_{\text{stent}}\left(\mathrm{FA}=15^{{}^{\circ}}\right)}=\frac{1}{1-\beta }$. Therefore, another measurement was performed with the adapted flip angle as well as an increased number of averages of ${\mathrm{av}}_{\text{stent}}=\frac{1}{{\left(1-\beta \right)}^{2}}\cdot {\mathrm{av}}_{\mathrm{ref}}$ to achieve the same intraluminal SNR as for the reference measurement without the stent.

In this work, *β* is expressed in %, as are absolute differences between shielding values *β*_k_–*β*_l_. When comparing multiple values, the maximum absolute difference in *β*, Δ*β*_max_ = abs(max(*β*_k_–*β*_l_)) [%], is indicated. Relative differences are explicitly mentioned when used.

## 3 Results

### 3.1 Linearly Polarized *B*_1_ : Circular and Rectangular Loops

**FIGURE 3:**
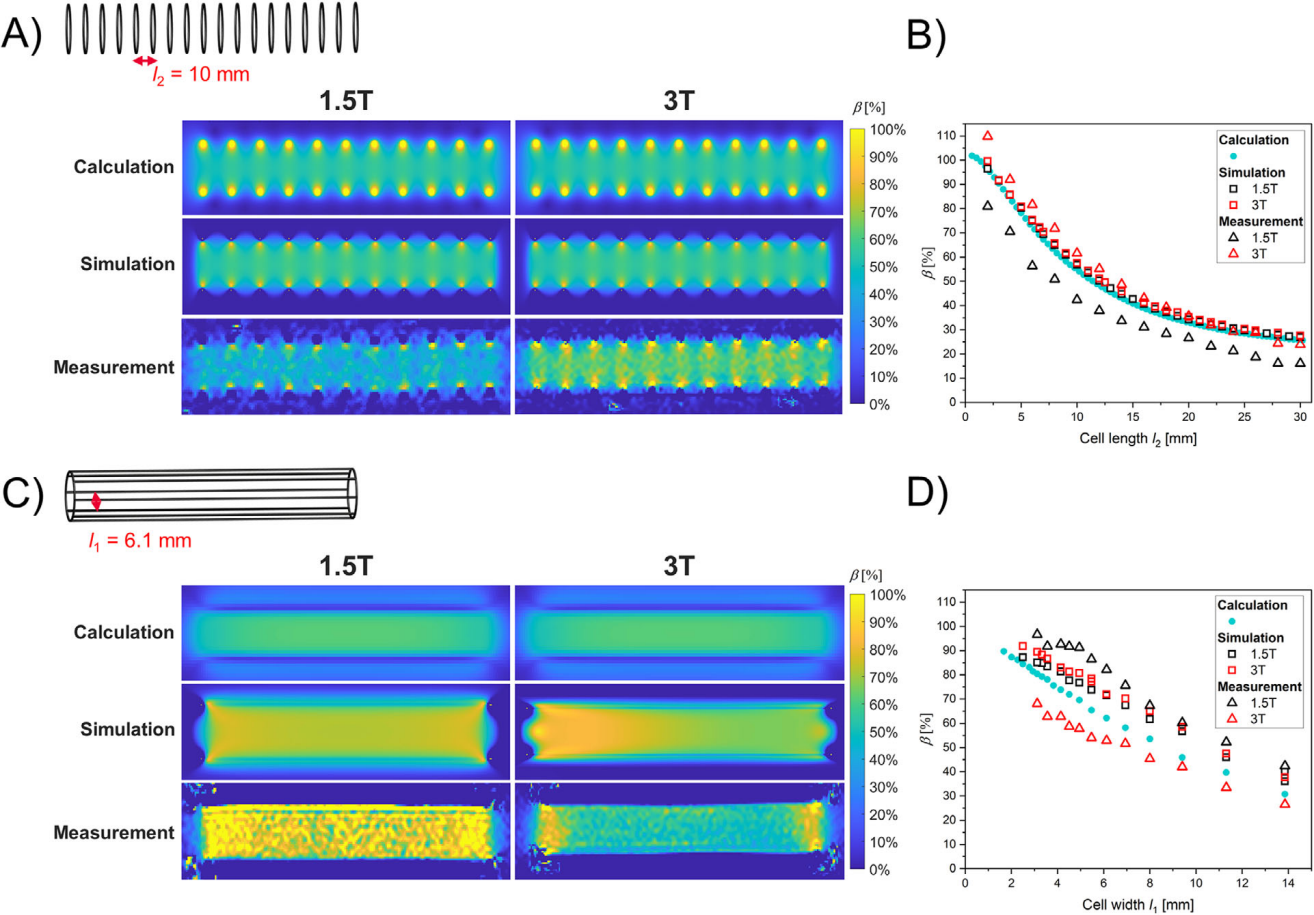
(A,C) Maps of *β* of circular loops (*l*_2_ = 10 mm) and rectangular loops (*N* = 8) for calculations, simulation and measurement at 1.5 T and (left) and 3 T (right). (B,D) Mean values of *β* in a central ROI at 1.5 T (black) and 3 T (red) versus the cell length l_2_ and the cell width *l*_1_.

Shielding maps of calculated, simulated and measured circular loops and rectangular loops orthogonal to *B*_0_ are exemplarily shown for *l*_2_ = 10 mm (Figure 3A) and *N* = 8 (*l*_1_ = 6.1 mm, Figure 3C). All maps show a similar shielding pattern for both, circular and rectangular loops. *β* of all circular (Figure 3B) and rectangular loops (Figure 3D) is plotted versus cell length *l*_2_ and cell width *l*_1_, respectively. Shielding decreases with increasing *l*_1_ or *l*_2_ for all loops. For rectangular loops, the decrease deviates from linearity for high *l*_1_. For circular loops, *β* decreases with 1/*l*_2_ for high *l*_2_. Calculated *β* absolutely deviates by < −3.3% for all *l*_2_ relative to the simulated *β* of circular loops. For all rectangular loops, Δ*β*_max_ (= abs(max(*β*_sim_ *–β*_calc_))) is 10% between calculated and simulated *β*. Maximum differences between measurement and simulation are −20% and 10% for circular loops, and 11% and −20% for rectangular loops at 1.5 and 3 T, respectively. For the rectangular loop, the simulated shielding map of 1.5 T has a homogeneous pattern whereas the shielding at 3 T decreases along the longitudinal axis. Here, the relative differences in *β* between 1.5 and 3 T are up to 4.1% for all *l*_1_. Results of the simulations of a model with rectangular loops and varying frequencies can be found in Figure S3. The profiles of *β* along the long axis of the stents show an increasing variation with increasing frequencies (5% at 63.8 MHz and 30% at 123 MHz).

The different lengths of *L* = 8 and 6 cm have negligible influence on the shielding (Figure S4): all circular loops and rectangular loops show a Δ*β*_max_ of 5% and 10%, respectively. The differences in *β* were higher in the simulations (8%) than in the calculations (3%).

### 3.2 Shielding of Rectangular Loops

**FIGURE 4:**
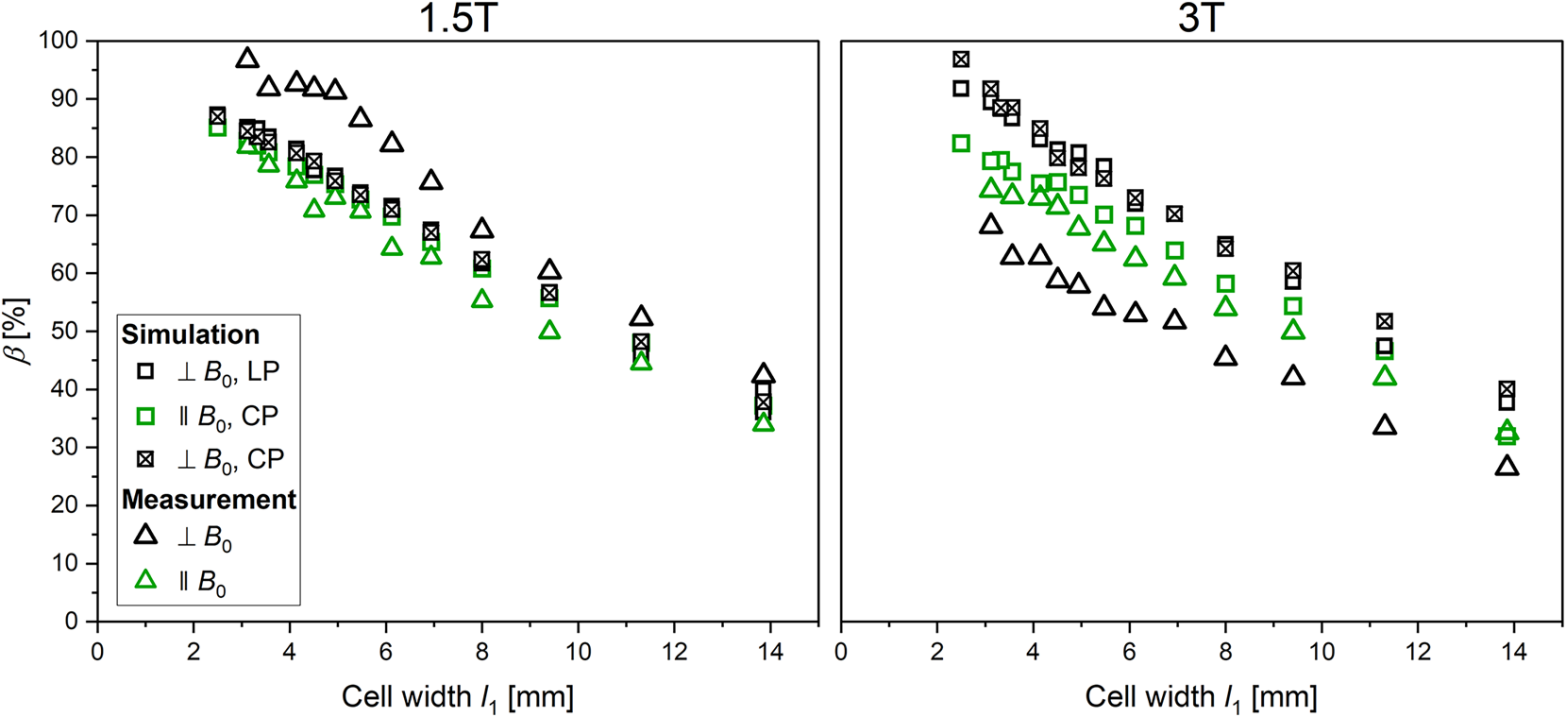
Measured (triangle) and simulated (square) values of *β* of rectangular loops versus the cell width *l*_1_ positioned parallel to *B*_0_ and orthogonal to *B*_0_. LP: linearly polarized *B*_1*y*_, CP: circularly polarized (*B*_1*x*_ and *B*_1*y*_).

Plots of simulated and measured *β* are shown in Figure 4 of all rectangular loops positioned parallel and orthogonal to *B*_0_ and for both, a circularly and linearly polarized field. For all simulated *β*, Δ*β*_max_ is 6% / 15% at 1.5 T/3 T, respectively. In the simulations with loops parallel to *B*_0_, the curves of *β* versus *l*_1_ are similar at 1.5 and 3 T (Δ*β*_max_ of 5%); for loops orthogonal to *B*_0_, Δ*β*_max_ between circularly and linearly polarized is 4.2% / 2% at 3 T/1.5 T, respectively. Δ*β*_max_ is 7% between measured and simulated rectangular loops, oriented parallel to *B*_0_. For measured rectangular loops parallel to *B*_0_, Δ*β*_max_ is 8% between 1.5 and 3 T.

### 3.3 Grid Stents and Composite Loops

**FIGURE 5:**
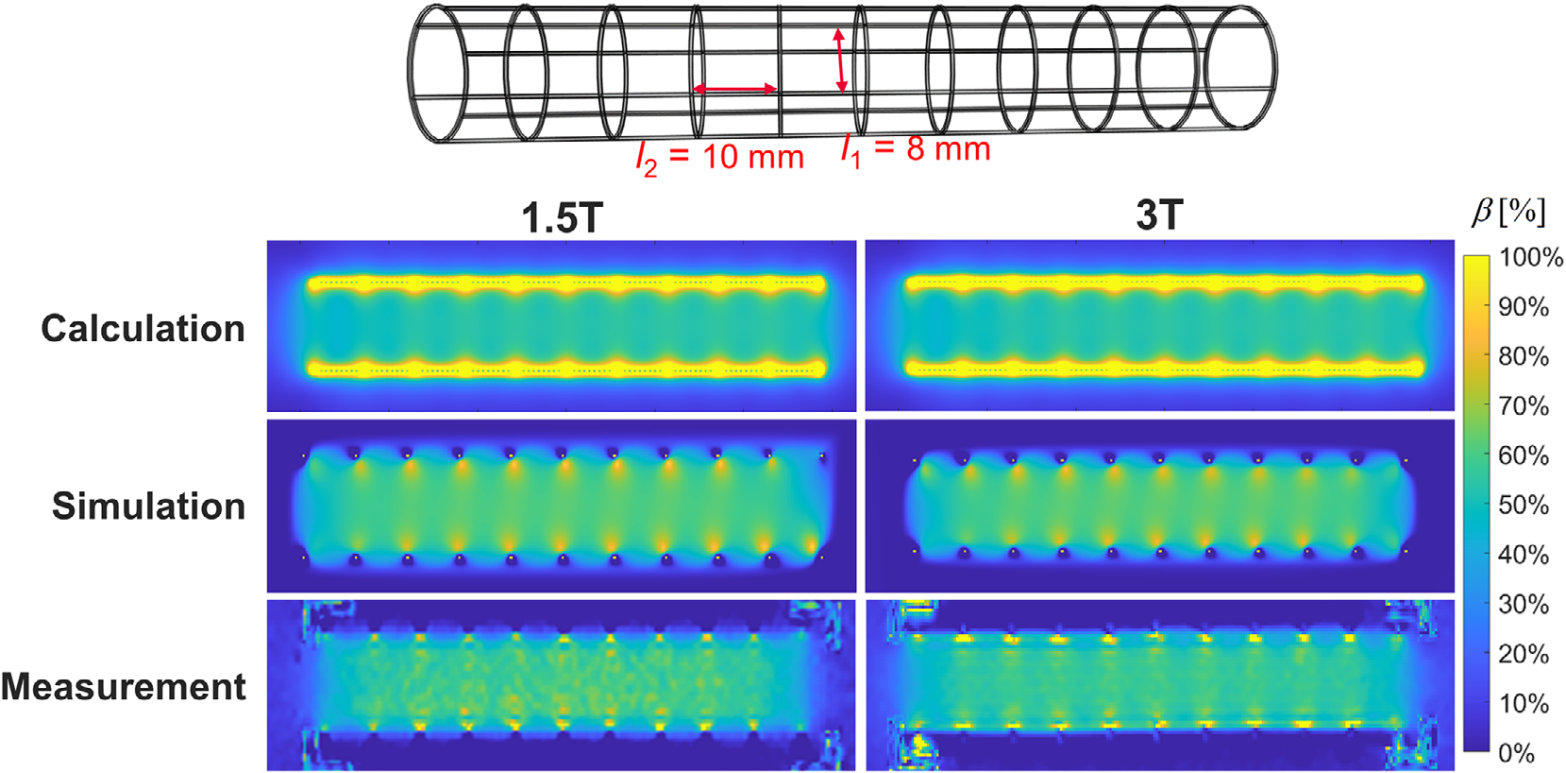
Maps of *β* of simulated and measured grid stents with [*N*, *l*_2_] = [6, 10 mm] in comparison to the calculated shielding of composite loops at 1.5 T (left) and 3 T (right).

The shielding of a grid stent ([*N*, *l*_2_] = [6, 10 mm]) is shown in Figure 5. Compared to simulations and measurements, the calculations show a stronger shielding at the longitudinal bars (*R* = 8 mm) whereas the pattern is similar inside the stents.

**FIGURE 6:**
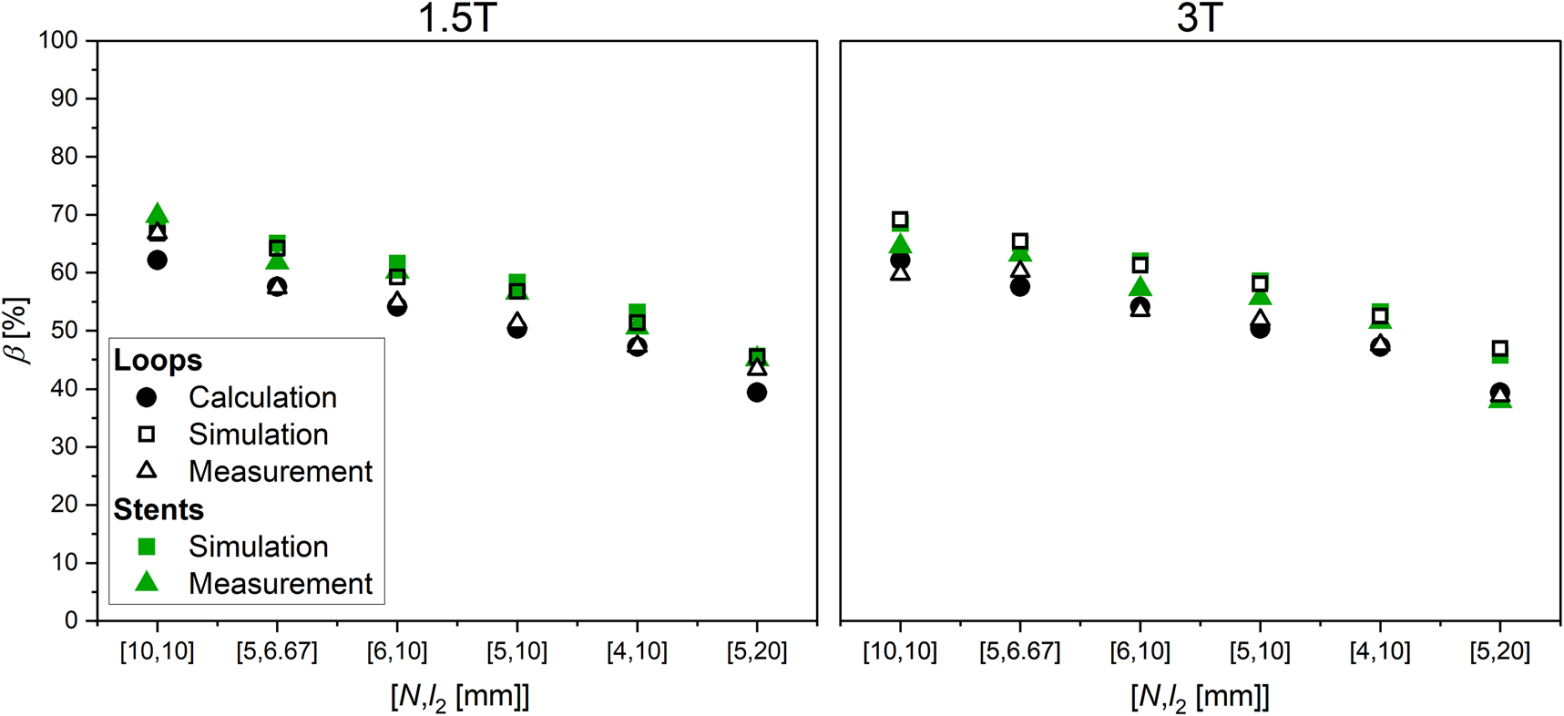
Values of *β* for simulated (square), measured (triangle) and calculated loops (loosen, black) as well as grid stents (solid, green) for 1.5 and 3 T.

In Figure 6 *β* is shown as a function of [*N*, *l*_2_] of all grid stents (“stents”) and composite loops (“loops”). Calculations, simulations, and measurements deviate only by Δ*β*_max_ = 10%. Δ*β*_max_ of grid stents and loops for both, 3 and 1.5 T, are 2% and 7% for simulations and measurements, respectively.

**FIGURE 7:**
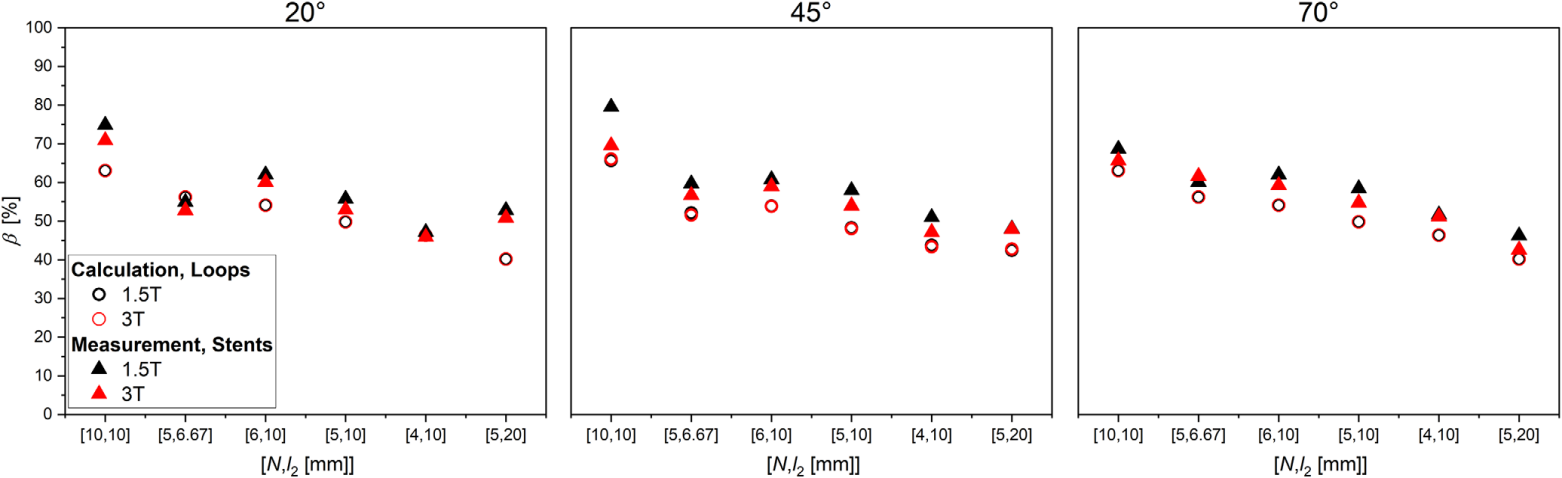
Values of *β* of loops (loosen) and grid stents with an angle of 20°, approx. 45° and 70° relative to *B*_0_ for 1.5 T (black) and 3 T (red), respectively.

Orienting the grid stents with an angle relative to *B*_0_, Δ*β*_max_ increased to 14% and 10% for 1.5 and 3 T, respectively, compared to the calculated shielding of composite loops (Figure 7).

### 3.4 Grid Stents and Commercial Laser‐Cut Venous Stents

**FIGURE 8:**
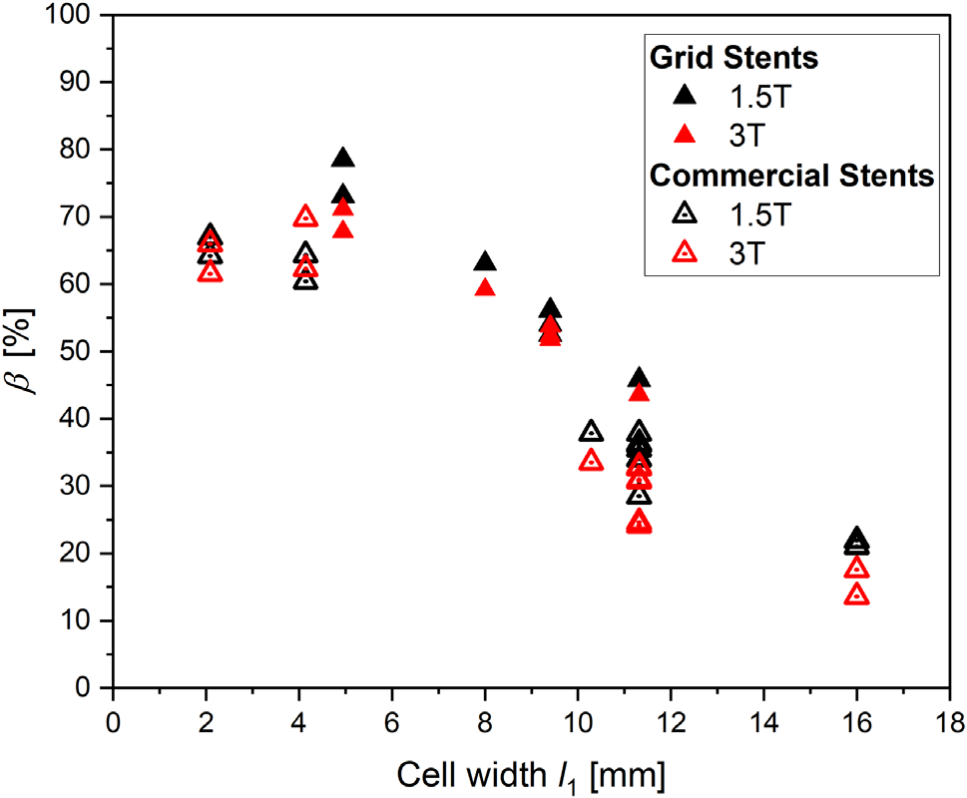
Values of *β* of commercial stents and stent models positioned parallel to *B*_0_ for 1.5 T (black) and 3 T (red).

In Figure 8, *β* is presented as a function of *l*_1_ of all grid stents and commercial laser‐cut venous stents parallel to *B*_0_. A linear relationship on *l*_1_ is visible for both stent types for both field strengths. *β* is similar for both, grid and commercial stents, with Δ*β*_max_ of 10% between 1.5 and 3 T. However, *β* of all commercial stents is lower than that of grid stents (Δ*β*_max_ = 30%).

**FIGURE 9:**
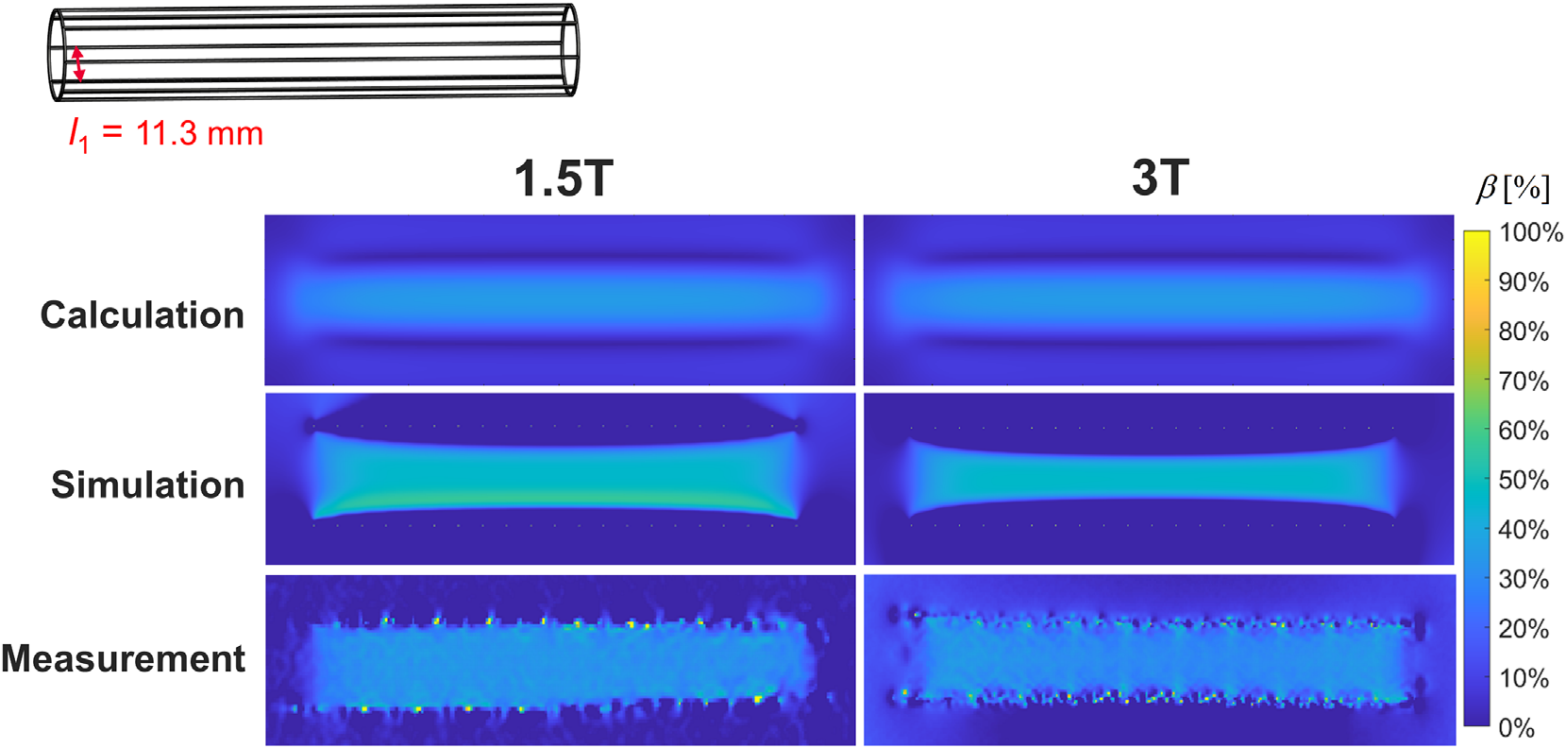
(A) Maps of *β* of one commercial laser‐cut venous stent ([*N*, *l*_2_] = [4, 6 mm]) for calculations, simulations and measurements at 1.5 T (left) and 3 T (right).

Measured shielding maps are shown in Figure 9 of one commercial stent with [*N*, *l*_2_] = [4, 6 mm] and *L* = 120 mm, oriented parallel to *B*_0_, at 3 and 1.5 T. The calculated and simulated maps were obtained with *ρ* = 0.05 mm. The simulated shielding is more inhomogeneous with a weaker shielding close to the stent surface. *β* values are 43.5% and 44% for the simulations, and 36% and 33.3% for the measurements at 1.5 and 3 T, respectively. Deviations from calculated *β* (35%) are < 9%.

**FIGURE 10:**
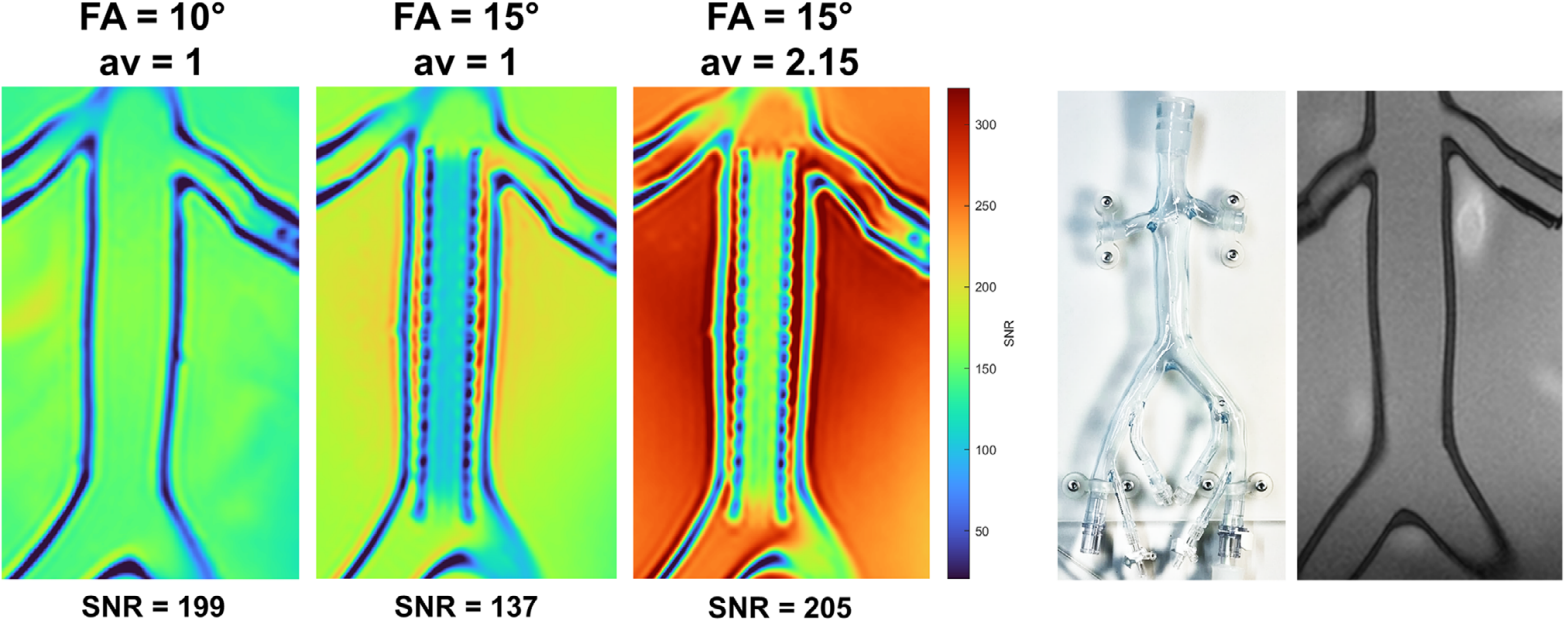
SNR maps with and without a commercial venous stent inserted in a venous phantom (right) for different FAs and averages. Intraluminal SNRs of the stent are given below.

The magnitude images of the commercial stent deployed in a venous phantom (Figure 10) showed $\frac{{\mathrm{SNR}}_{\mathrm{Ref}}\left(\mathrm{FA}=10^{{}^{\circ}},\;\mathrm{av}=1\right)}{{\mathrm{SNR}}_{\text{Stent}}\;\left(\mathrm{FA}=15^{{}^{\circ}},\;\mathrm{av}=1\right)}$ = $\frac{199}{137}$ = 1.45 (−2% for $\frac{1}{1-\beta }$ = 1.48). $\frac{{\mathrm{SNR}}_{\text{Stent}}\left(\mathrm{FA}=15^{{}^{\circ}},\;\mathrm{av}=1\right)}{{\mathrm{SNR}}_{\text{Stent}}\;\left(\mathrm{FA}=15^{{}^{\circ}},\;\mathrm{av}=2.15\right)}$ = $\frac{137}{205}$ = 0.67 (−3%) was in accordance with $\sqrt{\frac{{\mathrm{av}}_{1}}{{\mathrm{av}}_{2}}}$ = 0.68. This lead to $\frac{{\mathrm{SNR}}_{\text{Stent}}\;\left(\mathrm{FFA}=15^{{}^{\circ}},\;\mathrm{av}=2.15\right)}{\;{\mathrm{SNR}}_{\mathrm{Ref}}\left(\mathrm{FA}=10^{{}^{\circ}},\;\mathrm{av}=1\right)}$ = 1.03.

## 4 Discussion

This work provides a comprehensive study of the RF‐induced shielding of laser‐cut stents. A framework to calculate the shielding was realized including the cell geometry, and the results were compared to FDTD simulations and measurements at 1.5 and 3 T.

Previous studies only measured the shielding of stents in the circularly polarized *B*_1_‐field of the body coil. Thus, both *B*_1_ components interact with the stent structure and a clear relation between *l*_2_ or *l*_1_ and the shielding could not be established [14]. Separating circular and rectangular loops and investigating their shielding of the respective *B*_1_‐field components for the first time enabled establishing clear relations between the cell geometry and shielding under different stent orientations. Measuring the shielding of the individual *B*_1_ components would require special linearly polarized *T*_*x*_ coils. However, the results of this study demonstrate that the shielding of circularly polarized fields can be calculated from the results of the individual components. This can be adapted for stents positioned in any orientation to *B*_0_.

The work of Stollberger and Wach [37] first introduced the DAM method to map the *B*_1_+ field and it has been used in many studies since. They approximated the FLASH equation using long TR (*E*_1_ = 1) such that the influence of *T*_1_ can be neglected. Even though we use shorter TR in our study and compensate for that using the measured *T*_1_, the method remains sensitive to *B*_1_+ only (similar to Sung et al. [38]). The method is therefore intrinsically insensitive to receive homogeneities as the ratio of two magnitude images is calculated such that nonuniformities arising from the receive sensitivity are canceled out.

The inverse dependence of the shielding on *l*_2_ and *l*_1_ is in accordance with previous studies reporting lower intraluminal signal intensities for stents with smaller cell sizes [10]. Simulated *B*_1_ maps of Nitinol stents were presented by Guo et al. [19] that showed similar spatial profiles of *B*_1_ as in this work. The simulated shielding maps of rectangular loops showed a more inhomogeneous profile of *β* at 3 T compared to 1.5 T. Simulations with different frequencies furthermore indicated a continuous increase in inhomogeneity for increasing frequencies which may be attributed to standing wave effects. At 1.5 T, the wavelength is approximately 5× larger than *L* of the rectangular loops. At 3 T, the factor decreases to 2.5 (*λ* = 0.27 m); therefore, increasing the inhomogeneity of *B*_1_ inside the stents. The calculation model assumes quasi‐static fields such that this resonance effect is not accounted for. A linear dependence of *β* on *l*_1_ was also reported by Reiss et al. [14]. For circular loops, the measured *β* followed the monotonous decrease observed in simulations and calculations. For the rectangular loops, the measured *β* deviated from linearity which may be a result of variations in the manufacturing of the models: the longitudinal bars were not perfectly straight and positioned equidistantly along the circumference.

Measured values of *β* of rectangular loops at 1.5 T and of circular loops at 3 T were higher compared to simulation and calculation for all *l*_1_ and *l*_2_. Interpolating these results to *l*_2_, *l*_1_ → 0 mm would yield values of *β* > 100%, suggesting that the values were likely overestimated. The simulated and calculated loops were modeled as PEC. For the measured loops, a reduced shielding is expected due to the additional nonvanishing resistance (term: *RI*_i_) in Equation (7) that decreases the currents *I*_i_ induced in the loops [30]. Compared to the calculated values the skin effect reduces the effective strut radius and, possibly, the shielding: the skin depth is only 1–20 μm at 123.23 MHz, depending on the NiTi alloy, compared to the strut radius of 100 μm. This may explain the Δ*β*_max_ = 16% discrepancy between calculations and measurements (cf. Figure 9). Additionally, the nonvanishing resistance of the commercial stents could explain the reduced shielding in measurements compared to simulations. Furthermore, intensity relations close to 0.5 (cf. Equation 16) were obtained due to noise inside the loops. Thus, FA lower than 0.5 $\alpha_{\mathrm{BG}}$ and a shielding of 1 were calculated which resulted in increased β.

For the circular loops, the calculated and simulated *β* had a similar dependence on *l*_2_ with a maximum deviation of 3.3%. In contrast, for rectangular loops a systematic offset of approximately 10% was observed in the calculated *β*. The offset less likely results from the omission of the ceil (*N*/2) bar: simulating the rectangular loop (*N* = 5) with 4 bars resulted in only 1% less shielding. Furthermore, the values of *β* for an odd number of bars *N* did not deviate from the mean values of *β*(*N* − 1) and *β*(*N* + 1). Thus, omitting the ceil (*N*/2) bar was shown to provide a valid simplification of the calculation model. The systematic offset between calculated and simulated shielding values is more likely attributed to inaccuracies in calculating L and *M*. Additionally, the calculation model neglects the influence of the two circular loops located at *x* = −*L*/2 and *L*/2. The shielding of theses rings was only visible in simulated and measured shielding maps. However, as *B*_1ind_ of individual rings decreases with *B*_1ind_ ∝ 1/*x* ^2^ in the center, both rings contribute only *β* = 0.3% to the total shielding in the center and can thus be neglected.

This also explains, why the overall length *L* of the stent models with circular loops did not influence the shielding significantly. For the rectangular loops, however, the shielding was lower for the shorter stents with Δ*β*_max_ = 10%. This is consistent with previous findings reporting a minor relative shielding increase of approximately 7% for longer stents [14]. The self‐inductance of rectangular loops has a term that is linearly dependent on *L* (cf. Equation 12). However, as the mutual inductance has a nonlinear relationship with *L*, *I* = Φ/L only partially cancels this dependency.

The simulations of rectangular loops oriented orthogonal to *B*_0_ yielded similar *β* values for circularly and linearly polarized fields. This finding supports the assumption that *B*_1*x*_ is not shielded by the rectangular loops in this orientation. Consequently, the results demonstrate that Equation (18) can be used to calculate the shielding of measured rectangular loops, which also neglects the unshielded *B*_1*x*_.

Simulated shielding values of rectangular loops orthogonal to *B*_0_ were similar to those parallel to *B*_0_. The results confirm the intial assumptions made in Figure 1. Differences in *β* between both orientations, particularly, at 3 T, may result from standing wave‐effects induced by E‐field coupling, which was previously observed in linearly polarized fields at 3 T. At 1.5 T, the differences in *β* were minimal with 6%. The differences of *β* of measured rectangular loops between 1.5 and 3 T may be a result of differences in $\alpha_{\mathrm{BG}}$. Lower deviations of *β* from linearity on *l*_1_ were obtained applying higher FA as it increased the inner‐stent signal. It also reduced the differences in *β* between 1.5 and 3 T.

The shielding of a grid stent can be obtained by combining the shielding of circular and rectangular loops (Figures 5 and 6). The calculated shielding map (Figure 5) showed shielding ≫ 100% around the longitudinal bars which was not seen in simulations or measurements. An explanation may be the assumption of infinitesimally small wire radii (cf. *B*_1ind_ in Equation 8). At a small distance *u* from the current vector along longitudinal bars, *B*_1ind_ is inversely proportional to *u* ^2^, resulting in *β*(*x,y*) ≫ 100%. In the center, however, *β* is not influenced, since Δ*β*_max_ of 10% was obtained between the calculated model and measured and simulated data. Measured *β*_rect_ and *β*_circ_ at 1.5 and 3 T, respectively, were assumed to be overestimated and *β*_rect_ and *β*_circ_ at 3 and 1.5 T, respectively, underestimated. This was compensated by averaging ($\frac{1}{2}$(*β*_rect_ + *β*_circ_)) and Δ*β*_max_ of composite loops between 1.5 and 3 T reduced to 17%.

To calculate the shielding for arbitrary stent orientations in the coronal plane, the angle δ between the longitudinal axis and the *B*_0_ direction was introduced. The flux Φ through a circular loop then changes to $\pi R^{2}\left(\sin^{2}\left(\delta \right)B_{1x}\right)$. Thus, for arbitrary angles the shielding could be calculated using weighting factors of sin^2^(*δ*) and cos^2^(*δ*) for *B*_1*x*_ and *B*_1*y*_, respectively (i.e., using only the *B*_1_ components orthogonal to the loops). Figure 7 showed that the model can be used to calculate the shielding of stents in any orientation relative to *B*_0_, with Δ*β*_max_ less than 15%. Higher deviations between measurement and calculation were expected because the large deviations observed between measured and calculated rectangular loops (Figure 3) now contributed with: $\left(1{+\cos}^{2}\left(\delta \right)\right)$ *β*_rect_.

The comparison of grid stent models with commercial laser‐cut venous stents [14, 24] parallel to *B*_0_ yielded a difference of Δ*β*_max_ = 30%. The lower shielding of commercial stents likely results from the smaller strut radius (*ρ* = 0.05 mm) compared to *ρ* = 0.2 mm of the stent models: A lower diameter results in higher self‐inductance L, and thus lower induced current. A linear dependence on *l*_1_ was visible regardless of different *l*_2_, indicating that neither *B*_1*x*_ nor *B*_1*y*_ interact with the circular loops of a stent parallel to *B*_0_.

The lengths and cell sizes of the grid stents were chosen to mimic commercial laser‐cut venous stents. The model, however, can also be used to calculate the shielding of arterial stents [5] which often have smaller lengths and cell sizes [14]. For these stents the calculated *β* may even better agree with simulations and measurements as standing‐wave effects would be less prominent. As often more than one venous stent is applied, this may also affect the calculations which was already seen for certain stent configurations [39].

One major limitation of this work is the assumption of straight stents. In clinical practice, stents may be bent, for example, when they are positioned in the iliofemoral veins, which would require that the model needs to be adapted. For circular loops, the angle of each loop relative to *B*_1_ can be included in the calculation of Φ. For rectangular loops also the self‐inductance changes depending on the angle, which can calculated, for example, using the method presented by Venugopal et al. [31]. Overlapping of stents, however, makes the calculation of shielding difficult. For the circular loops, the size of *M* could be increased for both stents with different *l*_2_ and *R*_1_ and *R*_2_ and every *M*_12_ combination of *i*_1_ and *j*_1_ and *i*_2_ and *j*_2_ must be calculated. By changing the overlap of both stents, however, *l*_2_ between circular loops of both stents changes. To calculate *M*_ij_ of rectangular loops, the angles between two loops, one of each stent, must be known. Furthermore, the shift of rectangular loops from both stents relative to each other will change *M*_ij_. Calculating the shielding of overlapping stents was beyond the scope of this work and will be investigated in the future.

The knowledge of the shielding produced by a stent could be essential to adapt the applied FA and thus optimize the intraluminal signal. It was shown that increasing the FAs yields higher signal intensities inside the stents [11, 15, 17, 40]. For known *T*_1_ and TR, the FA yielding maximum signal (i.e., Ernst Angle) can be obtained from the FLASH equation. Aiming for the obtained FA to be the Ernst Angle, the applied FA could be increased by 1/(1 − *β*). By adapting the FA and number of averages in using the shielding, both the intraluminal flip angle and the SNR can be restored to their values without the stent. This allows maintaining image quality of established MR venography protocols in patients with stents; however, at the cost of increased acquisition times.

Commonly, contrast‐enhanced 3D GRE sequences with a FA of 10°–25° are used in clinical MR venography. Thus, increasing the FA based on the known shielding by the factor 1/(1 − *β*) is expected to be possible in most cases without exceeding SAR limits (e.g., 3.3‐fold adaptation of FA for the commercial stent with the highest shielding). This may be different for spin‐echo‐based sequences due to the 90° and 180° flip angle pulses for which an adaptation to compensate for shielding may indeed exceed SAR limits. However, even though techniques like ECG‐gated 3D TSE with phase subtraction to separately visualize arteries and veins provide spin echo‐based MR venography they suffer from poor image quality [41] and, thus, are less commonly applied. Furthermore, the substantially lower RF‐induced implant heating of GRE sequences compared to TSE renders GRE‐based sequences for MR venography in patients with stents more applicable [42]. Additionally, a recent study of in vivo stenting on pigs [43] showed that bSSFP, even though performed at low field strengths, produces strong artifacts rendering it less suitable for MR venography poststenting.

Bouillot et al. mentioned a difficult implementation of FA adaptation in clinical routine for every stent design and structure [12]. Even though bioresorbable stents exist that do not use metallic grid and, thus, do not reduce the intraluminal signal, the majority of stents still have electrically conducting structures [44]. This work presents a model to rapidly predict the shielding of a specific stent based on its cell geometry, which may help to improve imaging post‐stenting and monitoring of stent patency.

## Funding

This work was supported by the Federal Ministry of Education and Research (13GW0366).

## Supporting information


**Figure S1:** Soldered models for experiments. (A) Soldered circular loops, stacked with *l*_2_ = 6 mm. (B) Different rectangular loops with *N* = 4, 6, 9, 10, 12, 16. (C) Circular loops in the water phantom. (D) All grid stents and (E) their parameters.
**Figure S2:** Utilized commercial laser‐cut NiTi venous stents. The Sinus Obliquus stent has two different cell types. When two *L* are indicated, the number of cells along the long axis varies, but [*N*, *l*_2_] is identical. In that case, the picture of the larger stent is shown. *l*_2_ are approximate. Noninteger *N* result from shifted cells along the long axis.
**Figure S3:** Shielding profiles of rectangular loops for varying frequencies ranging from 63.8 to 250 MHz. With increasing frequency, the homogeneity of the shielding decreases.
**Figure S4:** Shielding of circular (A–C) and rectangular loops (D–F) for different lengths: *L* = 10 cm (square), 8 cm (circle), and 6 cm (triangle) for simulated 1.5 T, simulated 3 T, and calculations.

## Data Availability

The data that support the findings of this study are available from the corresponding author upon reasonable request.
