# Supplementary material for: RF‐Shielding of Laser‐Cut Venous Stents: Calculations, Simulations, and Experiments
Source: Magn Reson Med. 2025 Nov 30;95(4):2331–44. doi: 10.1002/mrm.70207 (PMC12850596; doi:10.1002/mrm.70207)
Supplement: Supplementary file 1 — Figure S1: Soldered models for experiments. (A) Soldered circular loops, stacked with l 2 = 6 mm. (B) Different rectangular loops with N = 4, 6, 9, 10, 12, 16. (C) Circular loops in the water phantom. (D) All grid stents and (E) their parameters. Figure S2: Utilized commercial laser‐cut NiTi venous stents. The Sinus Obliquus stent has two different cell types. When two L are indicated, the number of cells along the long axis varies, but [N, l 2] is identical. In that case, the picture of the larger stent is shown. l 2 are approximate. Noninteger N result from shifted cells along the long axis. Figure S3: Shielding profiles of rectangular loops for varying frequencies ranging from 63.8 to 250 MHz. With increasing frequency, the homogeneity of the shielding decreases. Figure S4: Shielding of circular (A–C) and rectangular loops (D–F) for different lengths: L = 10 cm (square), 8 cm (circle), and 6 cm (triangle) for simulated 1.5 T, simulated 3 T, and calculations. [file MRM-95-2331-s001.docx]

Supplementary Material


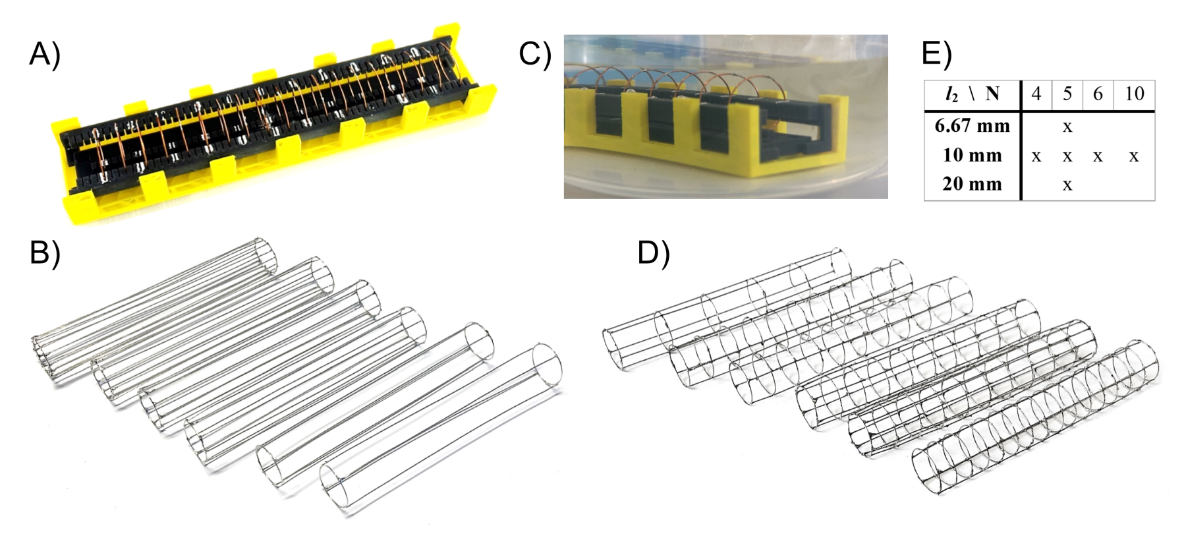


Figure S1 Soldered models for experiments. A) Soldered circular loops, stacked with l_2_ = 6 mm. B) Different rectangular loops with N = 4, 6, 9, 10, 12, 16. C) Circular loops in the water phantom. D) all grid stents and E) their parameters.


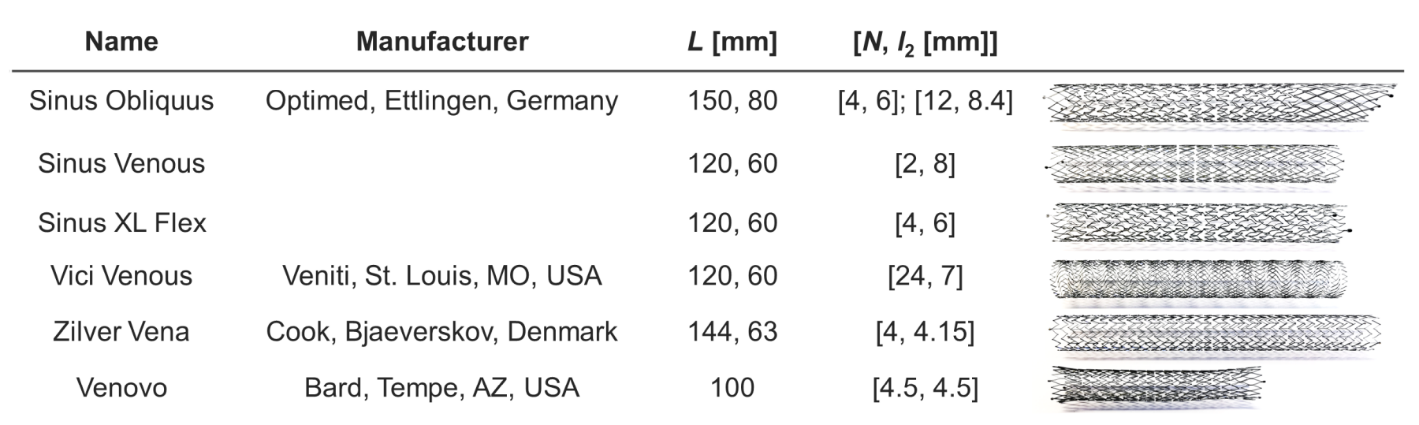


Figure S2 Utilized commercial laser-cut NiTi venous stents. The Sinus Obliquus stent has two different cell types. When two L are indicated, the number of cells along the long axis varies, but [N, l_2_] is identical. In that case, the picture of the larger stent is shown. l_2_ are approximate. Non-integer N result from shifted cells along the long axis.


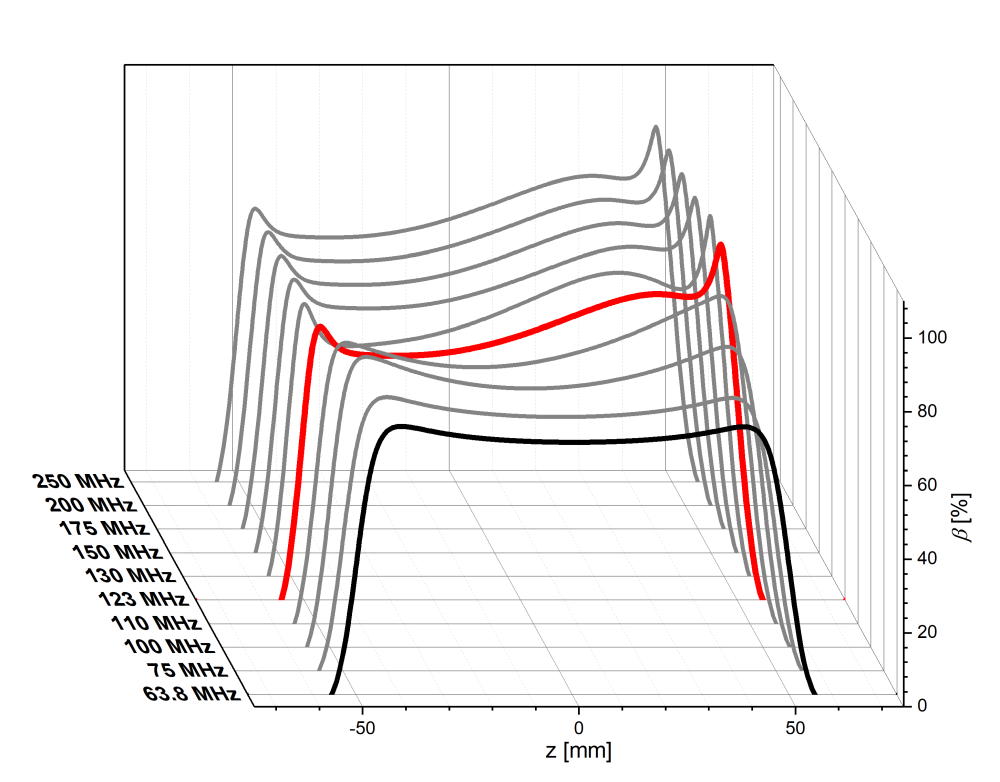


Figure S3 Shielding profiles of rectangular loops for varying frequencies ranging from 63.8 MHz – 250 MHz. With increasing frequency the homogeneity of the shielding decreases.


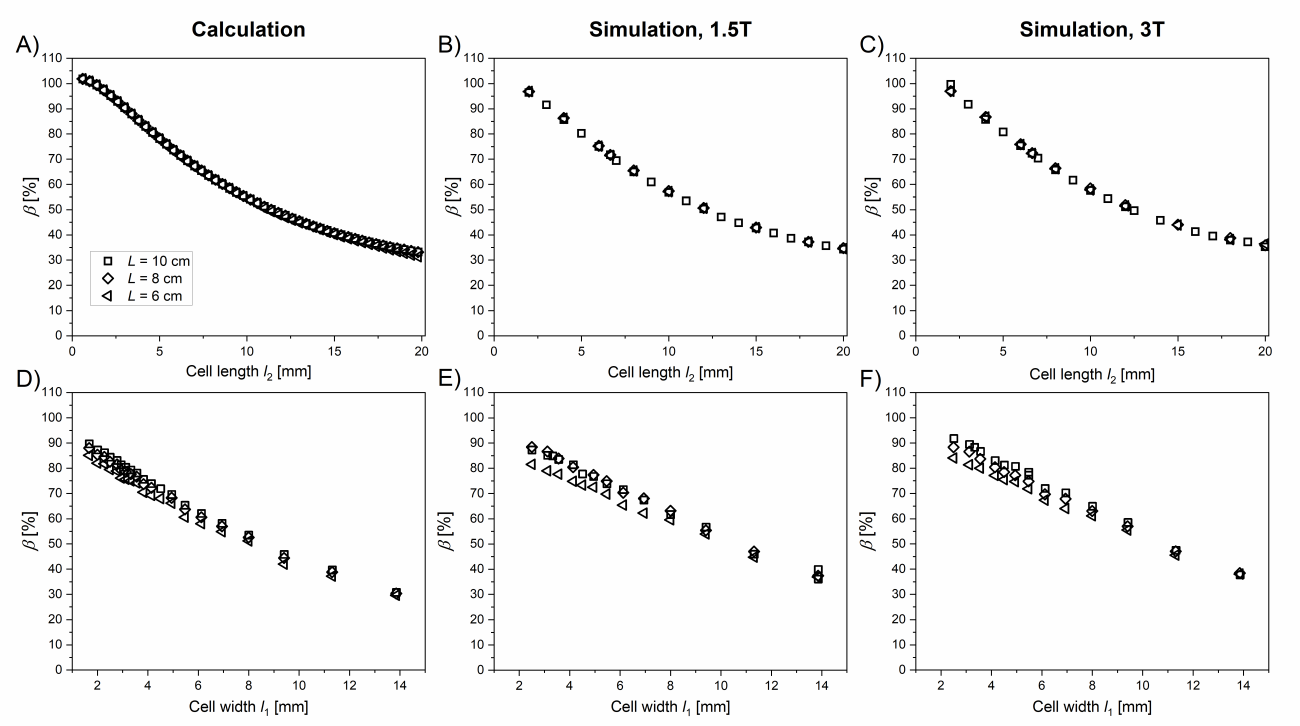


Figure S4 Shielding of circular (A,B,C) and rectangular loops (D,E,F) for different lengths: L = 10 cm (square), 8 cm (circle) and 6 cm (triangle) for simulated 1.5T, simulated 3T and calculations.
